# Quantitative classification of pancreatic atrophy: the İstanbul morphometric pancreatic atrophy classification based on CT and MRI measurements

**DOI:** 10.3389/fgstr.2026.1735281

**Published:** 2026-04-22

**Authors:** Fatih Öner Kaya, Esra Ümmühan Mermi, Alev Öztürk Günaldı, Haydar Kaan Karataş, Uğur Can Demir, Pınar Mert, Hüseyin Öztürk, Şule Sena Mazlum, Çağdaş Enginoğlu

**Affiliations:** 1 Department of Internal Medicine, Maltepe University Faculty of Medicine, Training and Research Hospital, İstanbul, Türkiye; 2 Özel Kartal Tıp Merkezi, İstanbul, Türkiye

**Keywords:** age-related changes, chronic pancreatitis, CT, imaging diagnostics, İstanbul classification, morphometry, MRI, pancreatic atrophy

## Abstract

**Background:**

Pancreatic atrophy (PA) is a progressive reduction in pancreatic parenchymal volume, accompanied by acinar cell loss, fibrosis, and fatty infiltration. It is an important radiological marker of glandular damage associated with physiological aging, chronic inflammatory diseases, and metabolic disturbances, such as diabetes mellitus and obesity. However, PA is still reported mainly using subjective terms, which leads to inter-observer variability and limits reliable clinical correlation and longitudinal follow-up.

**Purpose:**

This study aimed to introduce and validate the İstanbul Morphometric Pancreatic Atrophy Classification (IM-PAC), a four-tier morphometric grading system based on CT and MRI measurements, and to evaluate its association with graded CP severity features (CP).

**Methods:**

In this retrospective single-center study, 280 abdominal CT and MRI examinations performed in 2023 were analyzed. Pancreatic head, body, and tail thicknesses were measured at standardized anatomical landmarks. Chronic pancreatitis (CP)–related imaging features were graded using predefined ordinal severity scales for ductal abnormalities and MRI-defined fibrosis. Analyses used true modality-specific denominators (CT n = 168; MRI n = 112). Inter-observer reproducibility was assessed using intra-class correlation coefficients (ICC) and quadratic weighted Cohen’s kappa.

**Results:**

IM-PAC grades were distributed as Grade 0 (n = 54, 19.3%), Grade 1 (n = 119, 42.5%), Grade 2 (n = 93, 33.2%), and Grade 3 (n = 14, 5.0%). Increasing IM-PAC grade was significantly and monotonically associated with ductal abnormality severity (Spearman ρ = 0.52, p < 0.001) and MRI-defined fibrosis severity (ρ = 0.48, p < 0.001). Calcifications assessed in the CT subgroup (n = 168) showed progressive enrichment across grades (p < 0.001). Inter-observer agreement was excellent for thickness measurements (ICC = 0.89; 95% CI: 0.84–0.92) and substantial for ordinal ductal and fibrosis grading (weighted κ = 0.81 and 0.78, respectively).

**Conclusion:**

By integrating severity-based CP grading, IM-PAC provides a reproducible and clinically relevant morphometric framework for quantifying pancreatic atrophy.

## Introduction

Pancreatic atrophy (PA) is defined as a progressive loss of pancreatic glandular tissue, often accompanied by replacement with fibrous or adipose tissue, leading to reduced organ volume and functional capacity (1). On imaging, PA is characterized by decreased anteroposterior diameters, altered contours, and changes in attenuation or signal intensity.

Histologically, it is associated with acinar cell loss, ductal proliferation and metaplasia, chronic inflammatory infiltrates, and stromal fibrosis, features described in chronic pancreatitis (CP) and age-related pancreatic degeneration (1–5). An accurate definition of PA is challenging because it spans a continuum from physiological aging to advanced pathological atrophy.

In daily practice, radiologists frequently describe PA with broad qualitative terms such as “mild,” “moderate,” or “diffuse,” which show only moderate inter-observer agreement, with reported kappa values commonly below 0.6 (6–8). Quantitative approaches based on pancreatic volume or thickness (e.g., 20–40% volume loss relative to normative baselines) have been proposed, but thresholds differ across populations, modalities, and institutions, limiting their general use (8–11).

The etiological spectrum of PA is broad. Physiological atrophy accompanies advancing age due to cumulative cellular senescence, microvascular insufficiency, and hormonal changes, with pancreatic volume losses of 20–40% reported by the eighth decade of life (5, 9, 10, 12, 13). Pathological atrophy is a hallmark of CP, where repeated inflammatory attacks—often related to alcohol use, genetic mutations (e.g., PRSS1, CFTR), or idiopathic factors—lead to irreversible parenchymal destruction (1, 2, 14).

Metabolic disorders such as type 2 diabetes and obesity further contribute by inducing fatty infiltration, lipotoxicity, and insulin resistance (15–17). Additional causes include pancreatic ductal adenocarcinoma with obstructive atrophy, autoimmune pancreatitis within the spectrum of IgG4-related disease, genetic syndromes such as cystic fibrosis, Shwachman–Diamond syndrome, and Johanson–Blizzard syndrome, and iatrogenic factors including radiotherapy and certain medications (1, 14, 18).

Multiple imaging modalities can evaluate PA. Multiphase contrast-enhanced CT provides high spatial resolution and is well suited for morphometric assessment and detection of calcifications (6, 19–21). MRI offers superior tissue characterization, including evaluation of fibrosis (low T1 signal and delayed enhancement) and fat infiltration (high T1 signal or elevated proton-density fat fraction) (3, 11, 22). Ultrasound and endoscopic ultrasound (EUS) allow dynamic assessment and tissue sampling but are limited by operator dependence and restricted acoustic windows (7, 23). Emerging quantitative techniques such as MR elastography, CT volumetry, and radiomic analyses provide additional structural information but are not yet standardized for routine clinical use (8, 11, 24).

Although PA is commonly reported in abdominal imaging, its description remains largely qualitative. This subjectivity complicates comparison between studies, impairs risk stratification—for example, for exocrine insufficiency or malignancy—and may obscure the relationship between structural change and clinical outcome (6, 8, 25, 26). The development of high-resolution multidetector CT and multiparametric MRI now allows more precise and reproducible morphometric measurements. Several studies have emphasized the need for quantitative benchmarks, yet proposed thresholds for “normal” versus “atrophic” pancreas still vary widely and lack consistent external validation (3–5, 8–13, 15–17, 21, 22, 24, 27–33).

Normative adult pancreatic thicknesses are generally reported as approximately 23–28 mm for the head, 18–22 mm for the body, and 15–20 mm for the tail, with documented age- and sex-related variation (9, 10, 12, 27, 33). Multiple studies show progressive declines in pancreatic size with age, particularly after the fifth decade, and subtle sex differences that may reflect hormonal and body-composition effects (5, 10, 12). However, these values have not been integrated into a simple classification system suitable for everyday reporting. To address this gap, we developed the İstanbul Morphometric Pancreatic Atrophy Classification (IM-PAC), a four-tier, segment-based morphometric system built on anteroposterior thickness measurements of the pancreatic head, body, and tail. The objectives of this study were to: Define evidence-based IM-PAC thresholds using contemporary morphometric literature and our own cohort data; Examine the relationships between IM-PAC grades, demographic factors (age, sex), and metabolic comorbidities; and validate the clinical relevance of IM-PAC by assessing its association with established imaging markers of CP.

Normative values underlying IM-PAC were derived from a focused review of morphometric studies published between 2016 and 2025, summarized in **Table 1**. Four key studies were selected for methodological rigor, adequate sample size, precise anatomical definitions, and use of modern CT and MRI protocols. These studies reported convergent mean thickness values of approximately 25–26 mm for the head, 19–21 mm for the body, and 16–18 mm for the tail (9, 10, 12, 27). On this basis, we constructed IM-PAC thresholds using a hybrid approach that combined normative means with standard deviation (SD)–based stratification, allowing the grading system to capture meaningful departures from normal while remaining aligned with population-based evidence (5, 8, 9, 9–12, 27, 33).

**Table 1:** Normative morphometric reference summary.

Study	Year	Modality	Head (mm)	Body (mm)	Tail (mm)	n	Key notes
Wang et al. (11)	2024	MRI	25.5	20.2	17.1	450	Healthy adults; age-adjusted
Möller et al. (28)	2023	MRI/CT	25.8	19.5	16.8	320	Lifestyle and age effects
Treiber et al. (29)	2016	CT	24.7	18.9	16.4	280	Correlation with CP severity
Qin et al. (30)	2025	Mixed	25.2	19.8	17.0	500	Vanishing pancreas spectrum; imaging-based phenotyping

## Materials and methods

### Study design and population

This single-center retrospective observational study included 280 consecutive adult patients (age 18–85 years; mean ± SD, 54 ± 13 years; 51% male, 49% female) who underwent contrast-enhanced abdominal CT or MRI between 1 January and 31 December 2023 at Maltepe University Training and Research Hospital, İstanbul, Türkiye. The cohort was designed to reflect real-world clinical practice. Indications for imaging were:

- Nonspecific abdominal pain (n = 126; 45%),
- Routine health screening (n = 70; 25%),
- Suspected hepatobiliary or gastrointestinal pathology (n = 42; 15%),
- Metabolic disorder evaluation such as diabetes follow-up (n = 28; 10%), and,
- Other indications not primarily related to the pancreas (n = 14; 5%). CT was mainly used in acute presentations or when calcifications were suspected, while MRI was preferred for soft-tissue and fibrosis evaluation. Exclusion criteria were defined to minimize confounding and included:

Known pancreatic neoplasms (e.g., adenocarcinoma, neuroendocrine tumors) confirmed by imaging or histology;

Acute pancreatitis, defined clinically (acute epigastric pain with elevated amylase/lipase >3× upper limit of normal) and radiologically (pancreatic enlargement, edema, peripancreatic fluid collections, necrosis);

Prior pancreatic surgery, including partial resections, total pancreatectomy, or pancreaticoduodenectomy (Whipple procedure);

Suboptimal image quality due to motion artifacts, inadequate contrast opacification, severe beam-hardening, or axial slice thickness >3 mm; and.

Lack of written informed consent for retrospective use of imaging data.

This study was approved by the Maltepe University Ethics Committee (protocol no.: 2023/10-15; October 15, 2023). All procedures were conducted in accordance with the Declaration of Helsinki. Before analysis, patient data were anonymized in the hospital’s picture archiving and communication system (PACS) by removing identifiers, such as names, dates of birth, and medical record numbers.

### Imaging technique and measurement

Imaging protocols followed institutional standards. CT examinations were performed on multidetector scanners (≥64-slice) with intravenous iodinated contrast (iohexol, 1.5 mL/kg), acquired in the portal venous phase and reconstructed as axial images with slice thickness ≤3 mm (19, 21). MRI studies were performed on 1.5T or 3T scanners and included T1-weighted and T2-weighted sequences, diffusion-weighted imaging, and dynamic gadolinium-enhanced sequences; additional sequences for fibrosis and fat quantification were obtained when available (8, 11, 22).

- Pancreatic thickness was measured on axial images perpendicular to the gland’s long axis at predefined anatomical landmarks:
- Head: within the duodenal C-loop.
- Body: at the portal-splenic vein confluence.
- Tail: proximal to the splenic hilum.
- Measurements were performed by an experienced abdominal radiologist blinded to clinical data. A second radiologist independently measured a subset of 40 cases to assess reproducibility.

An abdominal radiologist with 10 years of experience (A.Ö.G.) performed all primary measurements and was blinded to the demographic and clinical information. To assess inter-observer agreement, a second experienced radiologist (E.Ü.M., 8 years of experience) independently measured a randomly selected subset of 40 cases (14% of the cohort).

In patients with suspected CP, ancillary imaging features were recorded according to the Radiological Society of North America consensus guidelines (6, 18, 34, 35).

### Chronic pancreatitis feature severity grading

To improve reproducibility and reflect the spectrum of chronic structural damage, CP-related imaging features were evaluated using ordinal rather than binary grading.

MRI-based analyses were performed only in examinations with adequate MRCP and dynamic post-contrast sequences. Severity distributions were calculated using the true evaluable MRI denominator (n = 112).

### Ductal abnormality severity

Ductal morphology was primarily assessed on MRCP and high-resolution T2-weighted sequences (n= 112 evaluable MRI examinations).

Severity was operationally defined as follows:

None (0): Smooth main pancreatic duct (MPD) with no irregularity or side-branch ectasia.
Mild (1): Focal ductal irregularity or minimal caliber variation without upstream dilatation.
Moderate (2): Multifocal beading or segmental narrowing with mild upstream dilatation.
Severe (3): Diffuse irregularity with marked beading, multiple strictures, or significant upstream dilatation.

### MRI-defined fibrosis severity

Fibrosis was evaluated in MRI examinations with adequate dynamic post-contrast phases (arterial, portal venous, and delayed).

Severity definitions:

None (0): Homogeneous parenchymal enhancement with preserved T1 signal.
Mild (1): Focal low T1 signal or subtle delayed enhancement.
Moderate (2): Regional hypointensity with persistent delayed enhancement.
Severe (3): Diffuse low T1 signal with pronounced delayed enhancement consistent with advanced fibrotic remodeling.

Evaluability criteria required complete dynamic sequences without motion artifact.

### Inter-observer agreement for ordinal features

In a representative subset of 40 MRI examinations, ordinal grading of ductal abnormality and fibrosis was independently performed by two radiologists.

Agreement was assessed using quadratic-weighted Cohen’s kappa with 1,000 bootstrap resamples to derive 95% confidence intervals.

### IM-PAC classification system

IM-PAC was designed as a hybrid model that combines literature-derived normative values with empirical findings from this cohort. Initial thresholds were based on key CT and MRI morphometry studies (9, 10, 12, 27), which reported mean thickness values of approximately 25–26 mm (head), 19–21 mm (body), and 16–18 mm (tail) in healthy adults (9, 10, 12). These thresholds were informed by prior morphometric studies reporting an expected decline of approximately 0.5–1.0 mm per decade after age 40. In contrast, our cohort-based analysis demonstrated a more modest decline of approximately 0.3–0.4 mm per decade, as detailed in the Results section (33). The final IM-PAC grading system is presented in **Table 2**.

**Table 2:** İstanbul morphometric pancreatic atrophy classification (IM-PAC).

IM-PAC grade	Head thickness (mm)	Body thickness (mm)	Tail thickness (mm)	Interpretation/typical clinical implications
Grade 0 – Normal	≥ 25	≥ 20	≥ 18	Physiological glandular thickness. No specific intervention; routine follow-up if clinical risk factors are present.
Grade 1 – Mild Atrophy	20–24	16–19	14–17	1 SD below the normal mean. Often represents early age-related or metabolic change; lifestyle and metabolic risk factor control may be considered.
Grade 2 – Moderate Atrophy	15–19	12–15	10–13	2 SD below the normal mean. Suggests progressive glandular atrophy; evaluation for chronic pancreatitis or metabolic disease may be appropriate.
Grade 3 – Severe Atrophy	< 15	< 12	< 10	≥3 SD below the normal mean. High likelihood of advanced structural pancreatic damage; assessment of exocrine and endocrine pancreatic function is recommended.

In the primary analysis, these thresholds were applied without age or sex stratification to preserve simplicity and facilitate clinical use. Exploratory subgroup analyses evaluated possible modifications by age and comorbidity (3–5, 9, 10, 12, 27).

### Statistical analysis

All analyses were performed using Python version 3.11. The pandas library was used for data management, NumPy for numerical calculations, SciPy for nonparametric tests, and statsmodels for additional modeling. A biostatistician reviewed the statistical plan. Continuous variables were summarized as mean ± SD if normally distributed or as median and interquartile range (IQR) if skewed. Normality was assessed with the Shapiro–Wilk test. Between-group comparisons used the Mann–Whitney U test for two groups (e.g., sex differences) and the Kruskal–Wallis H test for three or more groups (e.g., across IM-PAC grades). Spearman’s rank correlation coefficient (ρ) was used to evaluate associations between age, pancreatic thicknesses, and cumulative CP features.

Categorical variables were compared using chi-square or Fisher’s exact tests as appropriate. Bootstrapped 95% confidence intervals (CIs) were obtained using 1,000 resampling iterations. A two-sided p-value <0.05 was considered statistically significant; Bonferroni correction was applied when multiple comparisons were performed. Sample size calculations based on pilot data indicated that 280 patients would provide >80% power (α = 0.05) to detect moderate correlations (|ρ| ≥ 0.30) between age and pancreatic thickness. Potential confounding effects of comorbidities such as type 2 diabetes and obesity were explored in prespecified subgroup analyses.

Measurement precision was 0.1 mm; changes of ≥1 mm were regarded as clinically relevant in this morphometric context. Sensitivity analyses were performed for modality-specific associations (e.g., IM-PAC vs. calcifications in the CT subgroup only; IM-PAC vs. fibrosis in the evaluable MRI only).

## Results

### Patient demographics and baseline characteristics

The study population represented a typical tertiary-care cohort with mixed indications for abdominal imaging. Baseline characteristics are summarized in **Table 3**.

**Table 3:** Patient characteristics and comorbidities (n = 280).

Characteristic	Value
Age, years (mean ± SD)	54 ± 13
Age distribution, n (%)	
18–39 years	56 (20%)
40–59 years	126 (45%)
60–85 years	98 (35%)
Sex, n (%)	
Male	143 (51%)
Female	137 (49%)
Body mass index, kg/m^2^ (mean ± SD)	27.4 ± 5.1
Imaging modality, n (%)	
CT	168 (60%)
MRI	112 (40%)
Primary imaging indication, n (%)	
Abdominal pain	126 (45%)
Routine check-up	70 (25%)
Gastrointestinal evaluation	42 (15%)
Metabolic disorder follow-up	28 (10%)
Other	14 (5%)
Key comorbidities, n (%)	
Type 2 diabetes	56 (20%)
Obesity (BMI >30 kg/m^2^)	42 (15%)
Hypertension	70 (25%)
Alcohol use >20 g/day	28 (10%)
Smoking history	56 (20%)
No major comorbidities	112 (40%)
Suspected pancreatic condition, n (%)	
None	245 (87.5%)
Chronic pancreatitis features are present	35 (12.5%)

Inter-observer agreement for pancreatic thickness measurements was excellent: the overall ICC for head, body, and tail measurements was 0.89 (95% CI: 0.84–0.92).

### Overall morphometric findings and IM-PAC distribution

Mean pancreatic thicknesses were:

Head: 24.9 ± 4.9 mm (median 25 mm, IQR 21–28 mm),
Body: 18.9 ± 4.4 mm (median 19 mm, IQR 16–22 mm),
Tail: 16.6 ± 4.2 mm (median 17 mm, IQR 14–19 mm).

Distributions were approximately normal, supporting the use of SD-based thresholds in IM-PAC.

Application of IM-PAC yielded the following grade distribution:

Grade 0 (Normal): 54 patients (19.3%),
Grade 1 (Mild atrophy): 119 patients (42.5%),
Grade 2 (Moderate atrophy): 93 patients (33.2%),
Grade 3 (Severe atrophy): 14 patients (5.0%).

One patient with borderline measurements was excluded from grade-based subgroup analyses. Mild and moderate atrophy (Grades 1–2) were predominant, consistent with the older age and comorbidity burden of the cohort.

Age-stratified analysis showed a clear trend towards higher grades in older patients: approximately 50% of patients older than 70 years were classified as Grade 2 or 3, compared with 10% among those younger than 40 years (p < 0.001).

### Correlations with age, sex, and metabolic comorbidities

Age correlated negatively with pancreatic thickness in all segments:

Head: ρ = –0.41 (95% CI: –0.51 to –0.30),
Body: ρ = –0.46 (95% CI: –0.55 to –0.36),
Tail: ρ = –0.43 (95% CI: –0.53 to –0.32); all p < 0.001.

These correlations correspond to an estimated decline of approximately 0.3–0.4 mm per decade after age 40.

Intersegmental correlations were positive and statistically significant:

- Head–body: ρ = 0.61,
- Head–tail: ρ = 0.52,
- Body–tail: ρ = 0.41; all p < 0.001.

This pattern supports a diffuse atrophic process rather than focal change, which is more characteristic of obstructive or neoplastic conditions (5, 18). Sex-related differences in thickness were small and not statistically significant (p > 0.05 for all segments).

There was a non-significant trend towards lower tail thickness in females compared with males (16.2 ± 4.1 mm vs. 17.0 ± 4.3 mm, p = 0.08), consistent with previous reports of subtle sex-related variation (9, 10).

Metabolic comorbidities influenced pancreatic morphology. Patients with type 2 diabetes had thinner pancreata than non-diabetics (head thickness: 22.5 ± 4.2 mm vs. 25.5 ± 5.0 mm, p < 0.01) (16, 17).

Obese patients (BMI >30 kg/m^2^) had lower body thickness (17.2 ± 3.9 mm) than non-obese individuals (19.3 ± 4.5 mm; p < 0.05). These findings are consistent with the concept that metabolic syndrome, lipomatosis, and insulin resistance contribute to structural remodeling of the pancreas (15, 17, 30, 36).

### Associations between IM-PAC and CP severity

CP-related structural changes were evaluated using ordinal severity grading for ductal abnormalities and MRI-defined fibrosis.

#### CT subgroup (n = 168)

In the CT subgroup (n = 168), the distribution of IM-PAC grades was:

Grade 0 (n = 32; 19.0%), Grade 1 (n = 72; 42.9%), Grade 2 (n = 55; 32.7%), and Grade 3 (n = 9; 5.4%).

Calcifications, a CT-specific imaging feature, demonstrated a significant increasing trend across IM-PAC grades (χ^2^ = 22.1, p < 0.001).

#### MRI subgroup (n = 112)

In the MRI subgroup (n = 112), the distribution was:

Grade 0 (n = 22; 19.6%), Grade 1 (n = 47; 42.0%), Grade 2 (n = 37; 33.0%), and Grade 3 (n = 6; 5.4%). The distribution of IM-PAC grades across CT and MRI subgroups is presented in **Table 4**.

**Table 4:** Distribution of chronic pancreatitis imaging features by IM-PAC grade using true modality-specific denominators.

IM-PAC grade	CT examinations (n=168)	MRI examinations (n=112)
Grade 0	32 (19.0%)	22 (19.6%)
Grade 1	72 (42.9%)	47 (42.0%)
Grade 2	55 (32.7%)	37 (33.0%)
Grade 3	9 (5.4%)	6 (5.4%)
Total	168 (100%)	112 (100%)

Percentages were calculated using observed modality-specific denominators (CT n = 168; MRI n = 112).

### Interobserver agreement (ordinal CP features)

Ductal abnormality severity increased progressively with IM-PAC grade (p for trend < 0.001; ρ =0.52).

Fibrosis severity likewise demonstrated a graded monotonic association (p for trend < 0.001; ρ = 0.48).

### Ductal abnormality severity (MRI)

### Interobserver reliability for ordinal imaging features

Ductal severity: κw = 0.81 (95% CI: 0.70–0.90).

Fibrosis severity: κw = 0.78 (95% CI: 0.66–0.88). Agreement interpreted as substantial to excellent.

### Sensitivity analyses

To confirm that associations were not driven by modality mix, additional analyses were performed. Detailed cross-tabulations of ductal abnormality severity and MRI-defined fibrosis severity across IM-PAC grades are provided in **Tables 5**, **6**.

**Table 5:** IM-PAC none mild moderate severe.

Grade 0	19	3	0	0
Grade 1	41	6	0	0
Grade 2	16	10	8	3
Grade 3	1	1	2	2

Trend analysis: p < 0.001.

Spearman correlation: ρ = 0.52, p < 0.001.

**Table 6:** MRI-defined fibrosis severity.

IM-PAC	None	Mild	Moderate	Severe
Grade 0	20	2	0	0
Grade 1	41	6	0	0
Grade 2	18	9	7	3
Grade 3	1	1	2	2

Trend: p < 0.001 Spearman ρ = 0.48.

The association between IM-PAC grade and calcifications remained significant in the CT subgroup (χ^2^ = 22.1, p < 0.001). Similarly, the association between IM-PAC grade and fibrosis severity remained significant in the MRI subgroup (χ^2^ = 25.6, p < 0.001).

### Composite CP severity index (exploratory)

A weighted composite index (0–8) was constructed: MPD dilatation (0/1).

Calcifications (0/1) Ductal severity (0–3).

Fibrosis severity (0–3).

IM-PAC grade correlated positively with composite severity (ρ = 0.56, p < 0.001).

## Discussion

This study introduces the İstanbul Morphometric Pancreatic Atrophy Classification (IM-PAC), a simple, segment-based, quantitative system for grading pancreatic atrophy using routine CT and MRI. IM-PAC was derived from current morphometric literature, refined with cohort data, and validated by its associations with age, metabolic comorbidities, and CP-specific imaging findings. By incorporating modality-dependent assessability in the reporting of CP features (e.g., calcifications on CT, ductal irregularity, and fibrosis on MRI), we ensure more accurate and robust correlations, addressing potential biases from modality mix.

Importantly, ordinal grading was treated as the primary analytic framework rather than a secondary refinement. This approach enhances reproducibility and aligns IM-PAC with contemporary imaging standards that emphasize severity stratification rather than binary feature detection.

### Relationship to previous morphometric studies and classifications

Our morphometric values are consistent with prior CT and MRI studies reporting similar mean thicknesses and age-related declines in pancreatic size (5, 9, 10, 12, 27). The negative correlation between age and pancreatic thickness (ρ ≈ = –0.4 to –0.5) is consistent with earlier work demonstrating gradual fibrofatty replacement and volume loss across the lifespan (4, 5, 9, 10, 12). Intersegmental correlations (ρ = 0.41–0.61) suggest that atrophy usually affects the gland diffusely, in contrast to focal volume loss that may be seen in neoplastic or obstructive lesions, including “vanishing pancreas” phenomena (5, 6, 18, 37).

Traditional classifications, such as the Cambridge system, primarily focus on ductal changes and do not quantify parenchymal atrophy in a standardized manner (3, 6, 8). Volumetric indices and radiomic analyses add more detail but are not widely available and require additional software and expertise (7, 8, 11, 21, 24). IM-PAC provides an intermediate solution: it relies on simple linear measurements obtained from routine CT or MRI examinations, yet quantifies parenchymal atrophy in a structured and reproducible manner. Our findings show that IM-PAC grades are strongly associated with CP-related imaging features, including ductal irregularity, calcifications, and MRI-defined fibrosis (3, 6, 7, 19, 22, 34, 35). This supports the idea that IM-PAC reflects the severity of chronic structural damage rather than merely capturing age or body size.

### Age, sex, and metabolic effects

Age-related changes in pancreatic morphology observed in this study are consistent with longitudinal studies showing a progressive reduction in pancreatic volume and increased fat infiltration with aging (5, 9, 10, 12, 15). The estimated decline of 0.3–0.4 mm per decade after age 40 is compatible with previously described age-related atrophy patterns (9, 10, 12).

The observed decline of 0.3–0.4 mm per decade in our cohort is slightly lower than previously reported estimates (0.5–1.0 mm/decade). This discrepancy may reflect population-specific characteristics, differences in imaging protocols, or the inclusion of metabolically heterogeneous patients, highlighting the importance of cohort-based calibration of morphometric thresholds (5, 10, 12).

Sex-related differences were minor and mostly not significant, although a trend towards thinner tails in females was observed. This is in line with previous work suggesting subtle sex-associated differences, potentially related to hormonal factors and variations in fat distribution rather than large structural differences (5, 9, 10, 27).

Metabolic comorbidities, especially type 2 diabetes and obesity, were associated with lower pancreatic thickness, supporting the concept that metabolic stress and lipomatosis contribute to structural atrophy (15–17, 29, 30).

These findings emphasize the importance of considering metabolic background when interpreting PA and suggest that IM-PAC could help distinguish between physiological aging and metabolically accelerated atrophy.

### Clinical implications

IM-PAC offers a standardized language for reporting pancreatic atrophy. Instead of non-specific phrases such as “mild diffuse atrophy,” a report can state: “IM-PAC Grade 2 atrophy, body-dominant, with intraductal calcifications and ductal irregularity, compatible with chronic pancreatitis; recommend evaluation for exocrine insufficiency and glycemic status.” This type of structured reporting can improve communication between radiologists and clinicians and may facilitate more consistent decision-making (6, 25, 34, 38). Higher IM-PAC grades are likely associated with an increased risk of exocrine pancreatic insufficiency and endocrine dysfunction, which are important determinants of morbidity in CP (1, 16, 25, 38, 39). IM-PAC could therefore guide the selection of patients for functional tests (e.g., fecal elastase, secretin-stimulated MRCP) and for early initiation of pancreatic enzyme replacement therapy or closer glycemic monitoring (16, 22, 25, 38, 39). Because IM-PAC is simple and reproducible, it is also well-suited to longitudinal studies and follow-up imaging, enabling objective tracking of disease progression. In research, IM-PAC can be used as a standardized metric of parenchymal atrophy across centers and imaging modalities. The system is also amenable to automation using computer-assisted measurement and could serve as a reference or training label for radiomic and deep learning applications (6, 8, 11, 21, 24).

### Strengths and limitations

**Figure 1:**
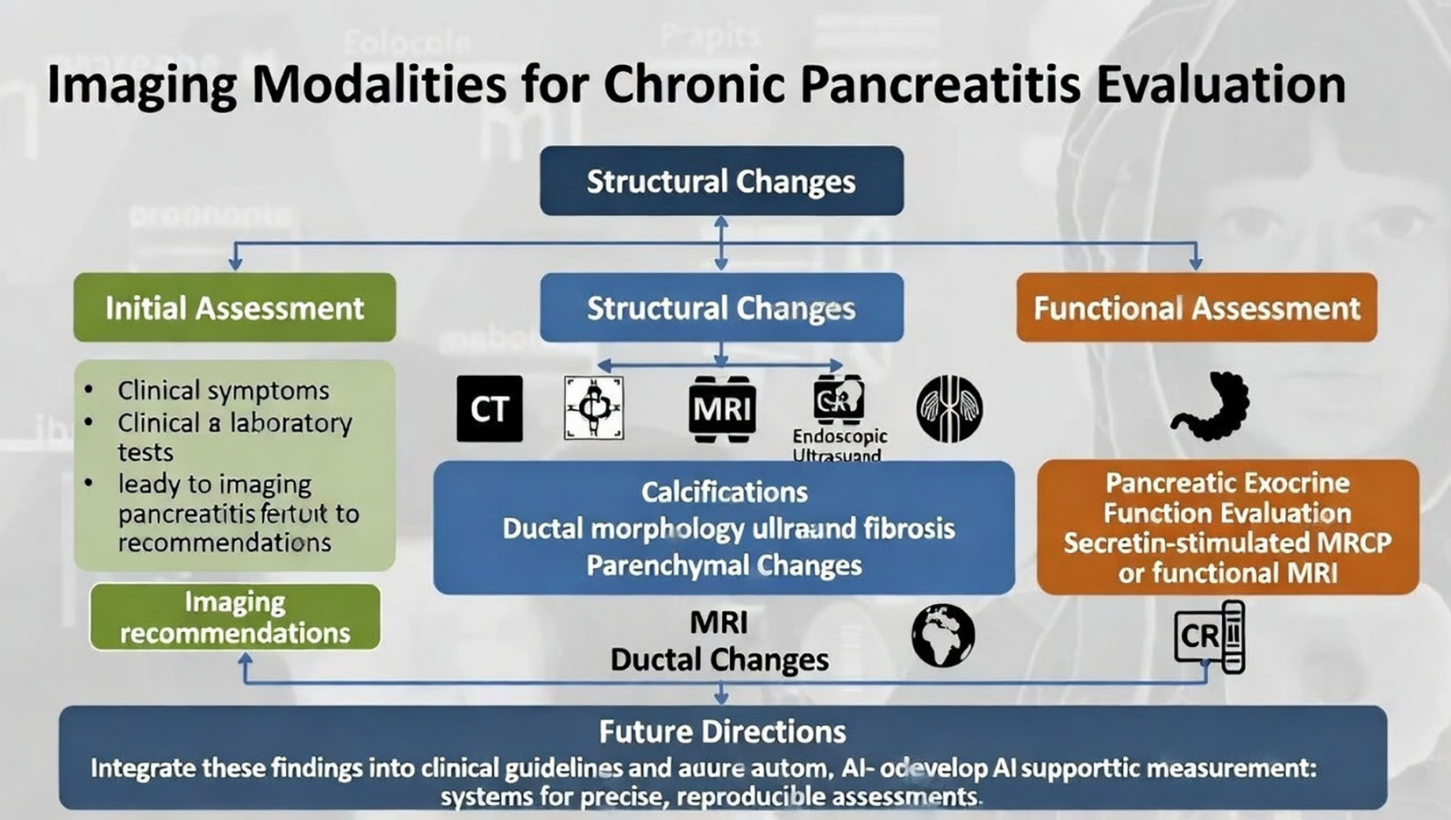
Imaging modalities for chronic pancreatitis evaluation.

Strengths of this study include a relatively large cohort, a real-world clinical setting, standardized measurement landmarks, and high inter-observer agreement. IM-PAC thresholds were grounded in contemporary literature and then validated empirically against CP-related imaging features, rather than being solely arbitrarily defined. The modality-specific reporting of CP features enhances the reliability of associations. Several limitations must be acknowledged. First, the retrospective single-center design may limit generalizability; external validation in different populations and with different imaging protocols is needed. Second, histopathological correlation was not available, so imaging-based atrophy and fibrosis could not be directly compared with microscopic findings (3). Third, IM-PAC uses linear measurements and does not capture the full three-dimensional complexity of the pancreas or subtle regional variations that volumetric and radiomic approaches can quantify (8, 11, 24, 32). Finally, although the influence of some comorbidities was explored, a full multivariable analysis adjusting for all relevant factors (e.g., alcohol intake, smoking, detailed metabolic indices) was beyond the scope of the current study (5, 13, 15–17). Future research should include prospective multicenter validation, integration with volumetric, fat fraction, and elastography data, and correlation of IM-PAC grades with functional outcomes such as exocrine insufficiency, diabetes progression, pain patterns, quality of life, and major clinical endpoints (11, 16, 25). Additionally, IM-PAC could be integrated into clinical guidelines, such as those from the American Pancreatic Association or RSNA, to standardize reporting. There is also potential for artificial intelligence-supported automatic measurement systems to enhance precision and reproducibility in routine practice. As illustrated in **Figure 1**, a proposed workflow for evaluating chronic pancreatitis features using various imaging modalities could facilitate this integration, with future directions including AI-automated measurements.

Although ordinal grading improves granularity, retrospective assessment may still underestimate subtle ductal irregularities. Prospective validation with standardized MRCP protocols is warranted (**Figure 1**).

Due to modality-dependent ascertainment, the use of non–age-adjusted thresholds, and the inherent limitations of retrospective imaging analyses, the present findings should be interpreted with caution and warrant further prospective, multicenter, multi-reader studies with standardized imaging protocols to confirm reproducibility and broader clinical generalizability.

## Conclusions

The İstanbul Morphometric Pancreatic Atrophy Classification (IM-PAC) is a practical, quantitative grading system based on simple anteroposterior thickness measurements of the pancreatic head, body, and tail on CT and MRI. In this cohort, IM-PAC:

Showed strong, biologically plausible correlations with age and metabolic comorbidities (Spearman ρ ranging from -0.21 to -0.16 for body and tail thicknesses, p < 0.01; ρ = 0.11 for overall grade, p = 0.058) and significant sex differences (Mann-Whitney U tests, p < 0.005 for all sites, with larger measurements in males);

Demonstrated robust associations with imaging features of chronic pancreatitis, including ductal irregularity, calcifications, and fibrosis, with modality-specific denominators ensuring accurate reporting; and.

Achieved excellent inter-observer reliability.

By replacing vague qualitative descriptors with reproducible segment-based thresholds, IM-PAC can improve consistency in radiological reporting, support objective longitudinal monitoring, and facilitate clinical and translational research in pancreatic disease. Broader implementation and external validation of IM-PAC may help integrate quantitative morphometry into routine practice and contribute to more precise risk stratification and individualized management of patients with pancreatic atrophy.

## Data Availability

The original contributions presented in the study are included in the article/supplementary material. Further inquiries can be directed to the corresponding author.
